# Elevated matrix metalloproteinase-7 expression promotes metastasis in human lung carcinoma

**DOI:** 10.1186/1477-7819-13-5

**Published:** 2015-01-14

**Authors:** Ji-Chang Han, Xian-Dong Li, Jin Du, Feng Xu, Yu-Ju Wei, Hong-Bing Li, Yi-Jie Zhang

**Affiliations:** Department of Respiration, Huaihe Hospital of Henan University, Ximen Street No. 115, Kaifeng, 475000 P.R China; Department of Gastroenterology, Huaihe Hospital of Henan University, Kaifeng, 475000 P.R China

**Keywords:** Matrix metalloproteinase 7, Protein expression, Lung cancer, Meta-analysis

## Abstract

**Background:**

Matrix metalloproteinase 7 (MMP-7) promotes tumor invasion and metastasis in several cancers. However, its role in lung cancer progression is understudied. In this study, we investigated the correlation between MMP-7 expression and lung cancer pathology.

**Methods:**

We searched the databases PubMed, Embase, Web of Science, Cochrane Library, CISCOM, CINAHL, China BioMedicine (CBM) and China National Knowledge Infrastructure (CNKI) for scientific literature relevant to MMP-7 and lung cancer. Carefully selected studies were pooled and ORs with 95% CI were calculated. Subgroup analyses and publication bias were analyzed to understand the retrieved data in greater detail. Version 12.0 STATA software was used for statistical analysis.

**Results:**

We retrieved a total of 121 studies through database searches. Finally, 14 cohort studies satisfied our inclusion/exclusion criteria, and these 14 studies, published between 2004 and 2012, were selected for meta-analysis to understand the influence of MMP-7 expression in lung cancer progression. Our results showed consistent differences in MMP-7 expression when comparisons were made between TNM I-II versus III-IV (OR = 1.82, 95% CI: 1.19 to 2.78, *P* = 0.006); histologic grade 1 to 2 versus 3 to 4 (OR = 1.67, 95% CI: 1.14 to 2.42, *P* = 0.008); and lymph node-negative versus lymph node-positive samples (OR = 2.81, 95% CI: 1.73 to 4.58, *P* <0.001), with significantly higher MMP-7 expression levels found in the more advanced stages. Subgroup analysis showed that age was not the factor influencing the associations between histologic grade, LN metastasis and MMP-7 expression in lung cancer patients, as both under 60 and over 60 age groups showed strong correlations (all *P* <0.05). However, when TNM staging was analyzed for its association with MMP-7 expression, only patients under age 60 showed a statistically significant correlation.

**Conclusions:**

Our meta-analysis results revealed that MMP-7 overexpression is associated with advanced TNM and histological grades, and is linked to aggressive LN metastasis in lung cancer patients; thus MMP-7 is a useful biomarker to assess the disease status in lung cancers.

## Background

Lung cancer is ranked at the top of all cancer-related deaths worldwide due to its high incidence rates, rapid malignant progression and current lack of specific and effective treatment strategies for lung cancers [1–3]. The clinical signs and symptoms of lung cancer present as persistent coughing, coughing up blood, wheezing, weight loss, fever, chest pain, bone pain, superior vena cava obstruction and difficulty in swallowing [4, 5]. Lung cancer remains the leading malignancy in males and is the second leading cancer in females among all cancers worldwide, and urban populations have higher morbidity rates than rural, due to air pollution [6, 7]. Lung cancer is associated with multiple risk factors, among which cigarette smoking is at the top, and other factors such as age, sex, excessive alcohol consumption, and air pollution are also major contributing factors in lung cancer [8, 9]. Despite our expanding knowledge of cancers and the latest improvements in clinical diagnosis and treatment, lung cancer patients have poor prognosis, with 5-year survival rates at only 9 to 20%. Thus, there is an urgent need to identify and evaluate the clinical value of factors relevant to lung cancer progression [10, 11]. Matrix metalloproteinase 7 (MMP-7) is one of the leading biomarkers in several cancers and has recently generated interest for its potential applications in lung cancer settings [12, 13].

MMP-7 is the smallest of the matrix metalloproteinases (MMPs) in size, with 28 kDa latent pro-form, and the mature form being 19 kDa [14]. MMP-7 is composed of a five-stranded β-sheet and three α-helices, with zinc-containing active domain, and other zinc and calcium ions necessary for structural stability [15]. As a typical member of the MMP family, MMP-7 has the ability to degrade extracellular matrix (ECM) components, such as elastin, type IV collagen, fibronectin, vitronectin, aggrecan and proteoglycans [16]. MMP-7 also exhibits proteinase activities against additional targets resulting in release of growth factors such as epidermal growth factor receptor (EGFR), heparin binding epidermal growth factor (HB-EGF) from the extracellular matrix, and in ectodomain shedding of cell-surface molecules, including Fas ligand and E-cadherin [17]. These activities of MMP-7 have important biological consequences relevant to tumor progression and metastasis. Not surprisingly, MMP-7 is linked to the disease progression in oral squamous cell carcinoma, prostate cancer, pancreatic cancer, colon cancer, breast cancer and non-small cell lung cancer [18–23]. A few studies have reported that MMP-7 shows a great promise as a biomarker to assess lung cancer proliferation, differentiation and metastasis [24, 25]. However, there are also studies with contrary results [26, 27]. Thus, we performed a systematic meta-analysis to evaluate the link between MMP-7 protein expression and the pathological features in lung cancer.

## Methods

### Data sources and keywords

We searched for studies published prior to 1 July 2014 that assessed the association between MMP-7 protein expression and the pathological features of lung cancer. Relevant studies were retrieved through information available from computerized databases such as PubMed, Embase, Web of Science, Cochrane Library, CISCOM, CINAHL, China BioMedicine (CBM) and China National Knowledge Infrastructure (CNKI), utilizing selected common keywords (‘matrix metalloproteinase 7’ or ‘MMP7 protein, human’ or ‘matrix metalloproteinase-7’ or ‘MMP-7’ or ‘MMP 7’ or ‘matrilysin’) and (‘lung neoplasms’ or ‘lung cancer’ or ‘lung carcinoma’ or ‘pulmonary carcinoma’ or ‘pulmonary cancer’ or ‘pulmonary neoplasms’ or ‘lung tumor’ or ‘pulmonary tumor’). No restriction was set to the language of the article. We also further manually scanned the bibliographies of relevant articles to identify additional potential relevant papers. If selected studies contained unclear data, the first authors were contacted for clarifications.

### Selection criteria

Published studies that were retrieved through database searches were further screened to meet the following selection criteria: (1) papers had to include lung cancer patients with different TNM stage, histologic grade, histology and LN metastasis status; (2) papers had to be human-associated clinical cohort studies that focused on the role of MMP-7 protein expression in the pathological features of lung cancer; (3) papers had to provide available data for MMP-7 protein expression; (4) papers had to report the adjusted odd ratios (ORs) at 95% confidence intervals (CI) for MMP-7 protein expression; and (5) papers had to report a minimum number of samples greater than 30. However, when the extracted studies had overlapping subjects of more than 50% in two or more papers, we selected the study that was the most comprehensive, and only the latest or most complete study was included when the extracted studies were published by the same authors.

### Data extraction

In order to reduce bias and enhance confidence in the data, two investigators separately extracted information from the retrieved papers according to the selection criteria and arrived at a consensus on all the items through discussion and reexamination. The following relevant data were extracted from eligible studies prospectively for the final meta-analysis: surname of first author, time of publication, ethnicity and country of publication, study type, study design, sample size, age and sex of subjects, source of samples, detection method for MMP-7 protein expression, MMP-7 protein expression in cases with different TNM stage, histologic grade, and histology or LN metastasis status. All authors approved the studies for their final inclusion in the meta-analysis.

### Quality assessment

To ensure that the study was high quality, two authors used a set of predefined criteria based on the Newcastle-Ottawa Scale (NOS) criteria to evaluate the studies independently [28]. The NOS criteria is scored based on three aspects: (1) subject selection: 0 to 4; (2) comparability of subject: 0 to 2; and (3) clinical outcome: 0 to 3. Total NOS scores range from 0 (lowest) to 9 (highest). According to the NOS scores, the included studies were classified into two levels: low quality (0 to 6), and high quality (7 to 9). Discrepancies on NOS scores of the enrolled articles were resolved by discussion and consultation with an additional reviewer.

### Statistical analysis

To calculate the effect size for each study, the summary ORs with 95% CI were used for TNM stage III-IV versus TNM stage I-II, histologic grade 1 to 2 versus histologic grade 3 to 4, adenocarcinoma versus squamous cell carcinoma, and with lymph node (LN) metastasis versus without LN metastasis categories of MMP-7 protein expression, using *Z* test. In order to supply quantitative evidence of all selected studies and minimize the variance of the summary ORs with 95% CI, we conducted the current statistical meta-analysis by utilizing a random-effects model (DerSimonian and Laird method) or a fixed effects model (Mantel-Haenszel method) of individual study results under the situation where data from independent studies were combined. A random effects model was applied when heterogeneity existed among studies, while a fixed effects model was applied when there was no statistical heterogeneity. The heterogeneity across the selected studies was evaluated by Cochran’s *Q*-statistic (*P* <0.05 was regarded as statistically significant) [29]. Due to low statistical power of Cochran’s *Q*-statistic, *I*^*2*^ test (0%, no heterogeneity; 100%, maximal heterogeneity) was also used to measure heterogeneity between studies [30]. The meta-regression and subgroup meta-analysis by detection method and country was conducted to explore potential effect modification. The one-way sensitivity analysis was performed through deleting single studies in our meta-analysis one by one to reflect the influence of the individual data set to the pooled ORs. The funnel plot was constructed to assess publication bias that might affect the validity of the estimates. The symmetry of the funnel plot was further assessed by Egger’s linear regression test [31]. To make sure that the results are credible and accurate, two investigators independently input all information in the STATA software, version 12.0 (Stata Corp, College Station, TX, USA) and arrived at similar results.

## Results

### Included studies

**Figure 1:**
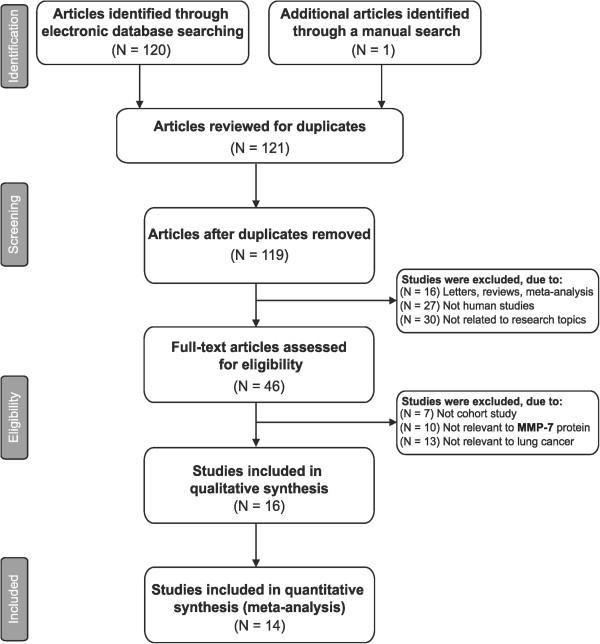
**Flow chart of study selection procedure.** Fourteen studies were included in this meta-analysis.

A flow chart of the study selection process is displayed in Figure 1. A total of 121 published studies were initially retrieved by electronic database searches, followed by manual search. We excluded duplicates (n = 2), letters, reviews or meta-analysis (n = 16), nonhuman studies (n = 27), studies not related to research topics (n = 30), noncase-controlled or cohort studies (n = 7), studies not relevant to MMP-7 protein studies (n = 10), studies not relevant to lung cancer (n = 13) and studies that did not supply enough information (n =2). Finally, a total of 14 cohort studies, published between 2004 and 2012, provided the necessary information about the correlation of protein expression of MMP-7 with the pathological features of lung cancer [17, 24, 25, 27, 32–41]. Demographic information on adult subjects with lung cancer, and other characteristics and methodological quality was extracted from the selected studies for our analysis. Thirteen studies were performed on Asian populations, and one study was on Caucasians. The 14 studies contained a total of 1,599 lung cancer patients with different TNM stages, histological grades, or LN metastasis status. The countries where the studies were performed were China (n = 12), Japan (n = 1), and Finland (n = 1). MMP-7 data in our present meta-analysis were all obtained from patient tissues. MMP-7 detection methods included streptavidin-peroxidase (SP, n = 8), and Non-SP (n = 6) including streptavidin-biotin peroxidase complex (SABC), SLAB, avidin-biotin-peroxidase complex (ABC) and APAAP. The baseline characteristics of the enrolled studies are listed in Table 1.

**Table 1:** **Baseline characteristics of the enrolled studies**

First author	Year	Country	Tumor	Sex (M/F)	Age (years)	Sample	Method	NOS score
Wang JX [39]	2012	China	56	41/15	58 (34 to 75)	Tissue	SP	6
Yamamoto T [27]	2012	Japan	78	54/24	64.7 ± 10.8	Tissue	SP	7
Bao WH [33]	2011	China	60	42/18	60 (36 to 73)	Tissue	SP	6
Ban YY [32]	2011	China	80	52/28	60 935 to 82)	Tissue	SP	7
Zhang Y [41]	2009	China	70	44/26	65 (48 to 79)	Tissue	SP	7
Song BH [38]	2009	China	66	52/14	59	Tissue	SP	6
Li JF [35]	2009	China	30	19/11	56 (40 to 82)	Tissue	SP	6
Xing YH [40]	2008	China	58	44/14	58 (36 to 78)	Tissue	Non-SP	6
Liu H [24]	2008	China	159	118/41	54 (39 to 74)	Tissue	Non-SP	8
Liu D [17]	2007	Japan	147	99/48	-	Tissue	Non-SP	7
Leinonen T [25]	2006	Finland	212	192/20	63 (42 to 78)	Tissue	Non-SP	8
Hsu CP [34]	2006	China	57	49/8	66.3 (36 to 84)	Tissue	Non-SP	6
Mei TH [37]	2004	China	74	45/29	54 (26 to 72)	Tissue	SP	7
Lin TS [36]	2004	China	452	361/91	-	Tissue	Non-SP	8

F, female; M, male; NOS, Newcastle-Ottawa Scale; SP, streptavidin-peroxidase.

### Association of matrix metalloproteinase 7 protein expression with pathological features of lung cancer

**Figure 2:**
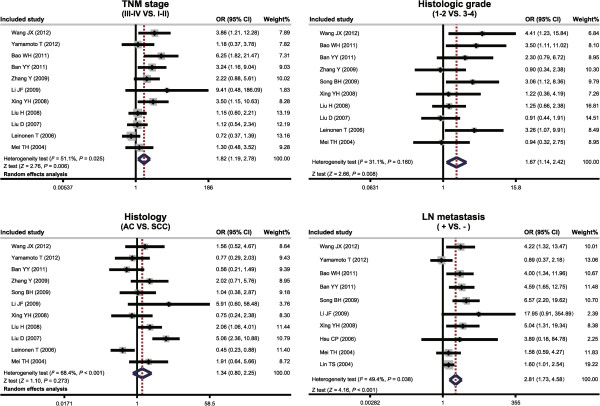
**Forest plots for the association between matrix metalloproteinase 7 (MMP-7) protein expression and the pathological characteristics of lung carcinoma.**

As shown in Figure 2, the major findings of the present meta-analysis revealed a significantly higher MMP-7 protein expression in lung cancer patients with TNM stage III-IV compared to patients with TNM stage I-II (OR = 1.82, 95% CI: 1.19-2.78, *P* = 0.006). However, Figure 2 also suggested MMP-7 protein expression in lung cancer patients with histologic grade 3 to 4 (3: moderately differentiated; 4: highly differentiated) was lower than that in patients with histologic grade 1 to 2 (1: undifferentiated; 2: poorly differentiated) (OR = 1.67, 95% CI: 1.14 to 2.42, *P* = 0.008). We did not detect a statistically significant difference in MMP-7 protein expression between adenocarcinoma patients and squamous cell carcinoma patients (*P* = 0.273), and MMP-7 protein expression in lung cancer patients with LN metastasis was significantly higher than that in patients without LN metastasis (OR = 2.81, 95% CI: 1.73-4.58, *P* <0.001).

**Figure 3:**
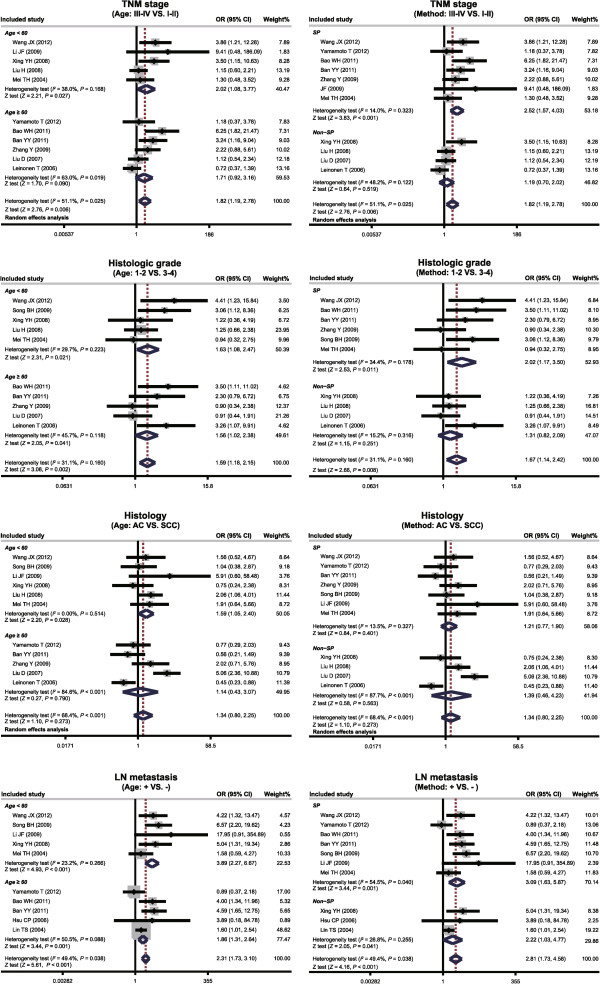
**Subgroup analyses of the association between matrix metalloproteinase 7 (MMP-7) protein expression and the pathological characteristics of lung carcinoma.**

Subgroup analysis based on age showed that higher MMP-7 protein expression was detected in lung cancer patients with TNM stage III-IV compared to TNM stage I-II in all patients under age 60 (OR = 2.43, 95% CI: 1.53 to 3.87, *P* <0.001), while there was no such statistically significant differences in patients over 60 years age (OR = 1.71, 95% CI = 0.92-3.16, *P* = 0.09). MMP-7 protein expression in lung cancer with histologic grade 3 to 4 was remarkably higher than histologic grade 1 to 2, which was not influenced by age (Age <60: OR = 1.63, 95% CI = 1.08 to 2.47, *P* = 0.021; Age ≥ 60: OR = 1.56, 95% CI = 1.02-2.38, *P* = 0.041) (as shown in Figure 3). It is also clear from Figure 3, based on histology, that MMP-7 protein expression in adenocarcinoma patients was significantly higher than squamous cell carcinoma patients, in patients under age 60 (OR = 1.59, 95% CI = 1.05 to 2.40, *P* = 0.028), but this difference was not statistically significant in patients over 60 years age (OR = 1.14, 95% CI = 0.43 to 3.07, *P* = 0.79). Interestingly, MMP-7 protein expression in lung cancer patients with lymph node metastasis was significantly higher than in lung cancer patients without lymphatic metastasis, which was not influenced by age (positive: OR = 3.89, 95% CI = 2.27 to 6.67, *P* <0.001; negative: OR = 1.86, 95% CI = 1.31 to 2.64, *P* <0.001) (Figure 3).

Further subgroup analysis based on MMP-7 detection methods revealed that MMP-7 protein expression in lung cancer patients with TNM stage III-IV expressed higher than the patients with TNM stage I-II by using SP (OR = 2.52, 95% CI: 1.57 to 4.03, *P* <0.001), while the difference of MMP-7 protein expression between lung cancer patients with TNM stage III-IV and patients with TNM stage I-II showed no statistical significance by using Non-SP (*P* = 0.519), as shown in Figure 3. A lower MMP-7 protein expression was detected by using SP in lung cancer patients with histologic grade 3 to 4 than that in patients with histologic grade 1 to 2 (OR = 2.02, 95% CI: 1.17 to 3.50, *P* = 0.011), while the difference of MMP-7 protein expression by using Non-SP between lung cancer patients with histologic grade 3 to 4 and patients with histologic grade 1 to 2 showed no statistical significance (*P* = 0.251) (as seen in Figure 3). The difference of MMP-7 protein expression between adenocarcinoma patients and squamous cell carcinoma patients has no obvious statistical significance whether by using SP or Non-SP (both *P* >0.05) (as shown in Figure 3). Furthermore, irrespective of SP or Non-SP, MMP-7 protein expression in lung cancer patients with LN metastasis was higher than patients without LN metastasis (both *P* <0.05), as shown in the Figure 3 subgroup analysis by detection method.

### Sensitivity analysis and publication bias

**Figure 4:**
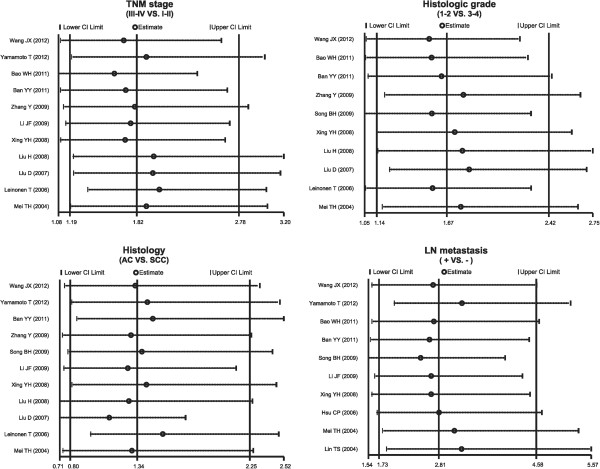
**Sensitivity analysis of the summary odds ratio coefficients for the association between matrix metalloproteinase 7 (MMP-7) protein expression and the pathological characteristics of lung carcinoma.**

**Figure 5:**
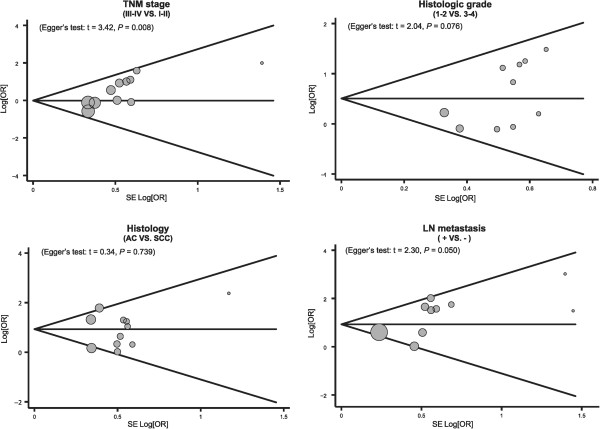
**Funnel plot of publication biases for the association between matrix metalloproteinase 7 (MMP-7) protein expression and the pathological characteristics of lung carcinoma.**

A sensitivity analysis was carried out to evaluate whether the present meta-analysis is stable. Each study enrolled in our meta-analysis was evaluated individually to reflect its effect on the significance of pooled ORs. The overall statistical significance did not change when any single study was omitted. Therefore, the current meta-analysis data is relatively stable and credible (Figure 4). The graphical funnel plots of those 14 studies for TNM stage III-IV versus TNM stage I-II for MMP-7 protein expression demonstrated evidence of obvious asymmetry, and Egger’s test displayed statistical evidence for publication bias (*P* <0.05) (Figure 5). However, the graphical funnel plots of the 14 studies for histologic grade 1 to 2 versus histologic grade 3 to 4, adenocarcinoma versus squamous cell carcinoma, with LN metastasis versus without LN metastasis for MMP-7 protein expression were symmetrical, and Egger's test showed no publication bias (all *P* >0.05) (Figure 5).

## Discussion

Our meta-analysis results reveal a strong link between MMP-7 overexpression and lung cancer progression. The main result of our analysis demonstrated a significant association between MMP-7 and TMN stages, histologic grade and LN metastasis of lung cancer but not between MMP-7 and histology. Glandular and ductal epithelial cells mainly produce MMP-7. MMP-7, when overexpressed in tumor cells, plays a central role in the progression of many tumors such as gastric cancer, esophageal cancer, colorectal cancer, liver cancer, renal cancer and pancreatic cancer and is a reliable indicator for high recurrence of cancer, poor prognosis and low rate of survival [42–44]. MMP-7 overexpression is reported to be associated with the TNM stage, histologic grade and LN metastasis of lung cancer. Our analysis shows that MMP-7 expression is higher in the III-IV stage than in the I-II stage and is overexpressed to a greater degree in the 1 to 2 histologic grade than in the 3 to 4 histologic grade, suggesting an important role of MMP-7 in the progression of lung cancer [17, 24]. Our multiple levels of analysis showed a consistent association between MMP-7 protein expression and LN metastasis, which suggest that MMP-7 mediated degradation of basement membrane could play a central role in lymphatic permeation and infiltrative growth of lung tumors [36]. MMP-7 overexpression could also regulate the proliferation of lung cancers through activation of EGFR and the production of mature HB-EGF to activate the receptor ErbB4. Wnt1 is involved in the regulation of MMP-7 expression, and therefore, Wnt signaling pathway plays a significant role in the lung cancers [17]. MMP-7 activity related to E-cadherin shedding could drive the accumulation of β-catenin in lung cancers overexpressing MMP-7, leading to the activation of multiple pathways involving dysregulated functions of both extracellular and intracellular components [45]. Thus, we conclude that MMP-7 overexpression is tightly linked with the TMN stages, histologic grade and LN metastasis of lung cancer, due to its direct ability for ECM degradation, tissue remodeling and tumor cell proliferation, and also indirectly influence lung cancer metastasis by the abnormal activation of the Wnt signaling pathway and the accumulation of β-catenin. Consistent with our analysis, Safranek *et al*. found higher MMP-7 protein expression in both adenocarcinoma and squamous lung carcinoma compared with benign lung tissue [13].

The age-stratified analysis demonstrated that the associations between histologic grade, LN metastasis, and MMP-7 overexpression are significant in lung cancer patients irrespective of age, while the association between TNM stages and MMP-7 overexpression is observed to be significant only in lung cancer patients under age 60. Thus, the MMP-7 overexpression pattern more closely reflects the tumor behavior than the current staging systems, indicating the role of MMP-7 as a potent biomarker for the pathological features of lung cancer.

Our meta-analysis sheds light on the association between MMP-7 protein expression and lung cancer progression, using a comprehensive and systematic approach based on previously published studies. All included studies in our meta-analysis were high-grade. While our meta-analysis has uncovered the advantages of MMP-7 based clinical evaluation, there does exist some limitations in the current meta-analysis. First, although we performed a methodological assessment of studies to avoid selection biases, there was significant heterogeneity among the 14 studies that may be attributed to the fact that the technique for detecting MMP-7 was not uniform among studies, or among the different ethnic backgrounds and lifestyles. Second, original data was unavailable for the present analysis, which might have had an impact on the stability of our overall results. Third, we could also not perform more precise calculation of ORs and further stratified analysis of potential heterogeneity that may affect the results. Fourth, some studies lacked information on age and detection method, and we examined one tumor biomarker expression in lung cancer cell lines. Therefore, the results of this study should act as a reliable primer for further focused studies, through prospective randomized controlled trials with integrity of information and more credible markers, to confirm our present finding and allow us to move toward clinical applications based on MMP-7.

## Conclusions

In summary, an increased expression of MMP-7 was found in the present meta-analysis to be strongly associated with advanced TNM stage, histologic grade, and aggressive LN metastasis in lung cancer patients. Based on our results, we propose that a MMP-7 specific inhibitor may prevent and treat invasion and metastasis of lung cancer. However, based on the above limitations, further in-depth studies are recommended to confirm our findings and to understand the exact role of MMP-7 in lung cancer progression.

## Authors’ information

Ji-Chang Han and Xian-Dong Li are both considered as first author.

## Abbreviations

ECM: extracellular matrix
NOS: Newcastle-Ottawa Scale
MMP-7: Matrix metalloproteinase 7
CBM: China BioMedicine
CNKI: China National Knowledge Infrastructure
MMPs: matrix metalloproteinases
ECM: extracellular matrix
EGFR: epidermal growth factor receptor
HB-EGF: heparin binding epidermal growth factor
CI: confidence intervals
SABC: streptavidin-biotin peroxidase complex
ABC: avidin-biotin-peroxidase complex.
